# Factors Associated with Prolonged Mechanical Ventilation in Late Repair of Tetralogy of Fallot

**DOI:** 10.1007/s00246-025-03786-9

**Published:** 2025-01-30

**Authors:** Amit Finkelstein, Rachel Sion-Sarid, Oren Zipper, Avital Mitler, Yair Erell

**Affiliations:** 1 School of Medicine, Tel Aviv University, Tel Aviv-Yafo, Israel; 2 Pediatric Intensive Care Department, Wolfson Medical Center, Holon, Israel; 3 Pediatric Cardiology Department, Wolfson Medical Center, Holon, Israel

**Keywords:** Tetralogy of Fallot, Late repair, Right ventricular outflow tract obstruction, Hypercyanotic spells, Invasive mechanical ventilation

## Abstract

Research establishing factors associated with duration of mechanical ventilation after Tetralogy of Fallot repair, is mainly based on population presenting at early infancy. There are fewer reports regarding repair after infancy, during childhood and preadolescence. To compare two groups of late TOF repair based on post-operative invasive mechanical ventilation duration and explore associations with pre-operative clinical markers of severity of right ventricular outflow tract obstruction. A single-center retrospective cohort study based on medical charts review of patients older than 12 months who underwent primary complete TOF repair between 2017 and 2023. Patients younger than 12 months or those who had distinct anatomical variants were excluded. Pre-operative characteristics reflecting the severity of right ventricular outflow tract obstruction were collected, including baseline oxygen saturation, the occurrence of hypercyanotic spells, maximum right ventricular outflow tract pressure gradient. Perioperative variables included repair type, cardiopulmonary bypass and aortic cross-clamp duration, and rates of residual lesions. 280 patients from 27 countries were screened; 181 remained eligible and were significantly older than previously reported. 129 (71%) were extubated early post-operatively, while 52 (29%) had a longer ventilation course. In a multivariate analysis, only baseline oxygen saturation and repair type maintained a significant association with length of mechanical ventilation. In distinctly older patients undergoing TOF repair, those with lower base line saturation undergoing a non-valve preserving repair are more likely to have a longer course of mechanical ventilation. Other clinical markers reflecting the severity of right ventricular outflow tract obstruction were not independently associated with mechanical ventilation duration.

**Supplementary Information:**

The online version contains supplementary material available at 10.1007/s00246-025-03786-9.

## Background

Tetralogy of Fallot (TOF) is the most common congenital cyanotic heart defect [1]. Current evidence supports complete surgical repair during infancy, optimally between 3 and 6 months of age when symptoms are not severe, and immediate earlier repair (complete or staged) when symptoms are severe [2]. As overall long-term transplant-free survival in repaired TOF is excellent [3], there is growing research focusing on immediate post-operative outcomes and complications in the pediatric intensive care unit (PICU), among which is the requirement for post-operative invasive mechanical ventilation (IMV), which impacts management aspects, other immediate outcomes, and complications [4–7].

In the last 3 decades, there has been a surge of research in mechanical ventilation in pediatric cardiac surgery and TOF specifically. Earlier clinical trials demonstrated favorable physiological outcomes with spontaneous breathing or, in select cases, with negative pressure ventilation [8–10]. Further work was published reporting the feasibility of early weaning from mechanical ventilation, from several hours post-operatively to immediately on the operating table, discussing anesthetic considerations, risk factors and predictive models for extubation failure, and general outcome measures [4, 11–16]. However, the larger cohorts and meta-analyses cover a wide range of age groups and cardiac defects, lacking granular physiological variables, limiting the ability to transfer conclusions to specific groups [17].

In more recent and specific TOF research, a standard definition for early extubation is lacking. It has been described in the range of immediate (in the operation room) and up to 72 h post-operatively [5]. Factors associated with prolonged IMV, specifically after TOF repair, have been studied primarily on the “conventional” population of infancy at the time of repair. Common pre-operative variables found to be associated with prolonged mechanical ventilation were younger age and lower baseline pulse oximetry saturation (SpO_2_) [5, 7]. Research focusing on late repair, after 12 months of age, with current surgical techniques, is less common and mainly reports long-term outcomes compared to the typical population [18–20].

Our cardiac surgery program has been caring for patients from low-income countries worldwide for the past 25 years, collaborating with *“Save a Child’s Heart”*, a non-profit organization dedicated to that purpose. Among them is a large population of TOF patients, uniquely brought to medical attention and care at a much older age than commonly reported. We sought to investigate factors associated with prolonged IMV in our specific patient population, as it is unclear if the current data from the general pediatric cardiac surgery and TOF population literature are applicable to these significantly older patients at the time of presentation and repair.

## Objectives

To describe the prevalence of post-operative prolonged IMV in a uniquely older patient population of TOF repair, and retrospectively determine if pre-operative clinical factors reflecting the severity of RVOT obstruction were significantly different in that group.

## Methods

This was a single-center retrospective cohort study based on medical chart review of surgical cases between 2017 and 2023. The study was approved by the hospital’s clinical research review board, and given its nature, the requirement for informed consent was waived (IRB Number 0089-23-WOMC). Patients who underwent complete TOF repair during the study period and at the time of surgery were older than 12 months, were eligible. We excluded patients who did not undergo primary complete repair, patients who, at the time of surgery, were less than 12 months old, patients with TOF pulmonic atresia, TOF absent pulmonic valve, TOF complete atrioventricular canal, and patients with evidence of major aortopulmonary collaterals (MAPCA’s). The main outcome variable was post-operative duration of IMV, categorically defined as prolonged (vs standard), if time to extubation was more than 24 h post-operatively or re-intubation occurred in the first 24 h post-extubation.

All surgeries during the study period were performed by one of two senior surgeons. Surgical approach (trans-atrial, valve preservation vs non-valve preservation) was chosen according to echocardiographic measurements, the ability to pass a -2 Z score matching sized probe through the pulmonary annulus, and degree of pulmonary valve dysplasia. In most cases, when a transannular patch augmentation was chosen, a polytetrafluoroethylene (PTFE) monocusp or bicuspid valve was recreated [21].

We searched the electronic/paper medical records for patients’ screening and data collection. Research variables included demographic and clinical characteristics, pre-operative SpO_2_, presence of hypercyanotic spells, RVOT maximum pressure gradient, and dimensions of the pulmonary valve and pulmonary arteries as measured by awake or minimally sedated pre-operative transthoracic echocardiography. Operative variables included cardiopulmonary bypass (CPB) and aortic cross-clamp duration, and type of surgical repair (valve preserving vs. non-valve-preserving). Post-operative variables included residual lesions, duration of post-operative IMV and re-intubation in the first 24 h post-extubation. Study data were collected and managed using REDCap electronic data capture tools hosted at our institution [22, 23].

### Statistical Analysis

Descriptive statistics for all demographic, baseline clinical characteristics, surgical, and post-operative data were calculated. Shapiro–Wilk test was used to evaluate the normal distribution of continuous variables. Means and standard deviations, or medians and interquartile ranges, were calculated accordingly. For categorical data, frequency counts and percentages were calculated. For univariate analysis, differences between standard IMV and prolonged IMV groups were analyzed using a *t*-test for continuous variables with normal distribution, Mann–Whitney *U* test for non-normal distribution, and Fisher’s exact test for categorical data. Results were considered significant if a two-sided *p*-value < 0.05. For multivariate analysis, a stepwise logistic regression was used on all significant outcomes from the univariate analysis. Receiver operating characteristic (ROC) analysis of the predicted probability was performed to assess the accuracy of the prediction model. Calculations were performed using JASP statistics computer software (JASP Team 2024, Version 0.18.3).

## Results

**Fig. 1:**
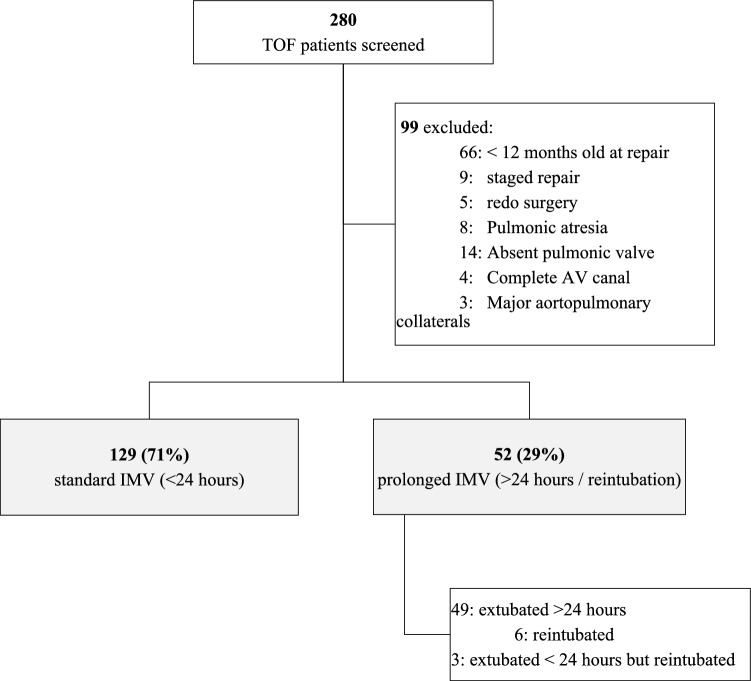
Flow diagram of study cohort

A medical records search between January 2017 and December 2023 resulted in 280 TOF repair patients arriving at our care from 27 countries in Africa, Asia, and Europe. Figure 1 details patients’ selection and exclusion. Of the remaining 181 patients in the cohort, 129 (71%) were extubated less than 24 h post-operatively (standard IMV group), while 52 (29%) were either extubated more than 24 h post-operatively or were reintubated in the first 24 h post-extubation (prolonged IMV group). In 45 (87%) cases, the primary reason for prolonged mechanical ventilation or extubation failure was cardiopulmonary, as listed in Table 1. Demographics, baseline clinical and surgical variables, and surgical outcomes of the two groups are presented in Table 2. Univariate analysis showed a statistically significant difference between the outcome groups in terms of pre-operative SpO_2_, pre-operative IMV, pulmonic valve Z value, CPB and aortic cross-clamp duration, and rates of valve preservation repair, residual pulmonic regurgitation (PR), delayed sternal closure and post-operative ECMO support. Pre-operative RVOT maximum pressure gradient did not reach a significant difference, nor did the rate of hypercyanotic spells, the general rate of additional cardiac lesions and the rate of post-operative residual VSD. The rate of pre-operative PFO/ASD was higher in the prolonged IMV group, as shown in Supplementary Table 1.

**Table 1:** Reasons for prolonged invasive mechanical ventilation

Reason	*n* (%)
Cardiopulmonary	45 (87)
Hemodynamic instability	40
Significant pulmonary edema	9
Significant pleural effusion	5
Ascites	2
Hypoxemia	1
Other	4 (7.5)
Redo for post-operative bleeding	1
Significant atelectasis	3
Not reported	3 (5.5)

**Table 2:** Patients’ characteristics

Variable^ab^	Full cohort *n* = 181	Standard IMV *n* = 129 (71%)	Prolonged IMV *n* = 52 (29%)	*P* value
Age (months)	50 (27, 82)	50 (29, 82)	49.5 (26, 80.75)	0.852
Weight (kg)	13.1 (10, 17)	13.4 (10.6, 17.5)	11.85 (9.7, 17)	0.193
Height (cm)	97 (80.4, 116)	98 (83, 117)	94.5 (77, 113)	0.348
BSA (m^2^)	0.6 (0.47, 0.75)	0.61 (0.49, 0.76)	0.57 (0.44, 0.73)	0.253
Males	108 (59.7)	80 (62)	28 (53.8)	0.320
Syndrome	14 (7.7)	9 (7)	5 (9.6)	0.548
Pre-operative SpO_2_ (%)	80 (72, 90)	83 (75, 90)	74 (61, 84.5)	< .001
RVOT max pressure gradient (mmHg)	73 (65, 82)	75 (67.5,85)	70 (64, 79)	0.053
Spells	70 (38.7)	45 (34.9)	25 (48.1)	0.129
Hemoglobin (g/dl)	16.6 (13.7, 19.4)	16.4 (13.5, 18.8)	17 (14.6, 20.8)	0.116
Pre-operative IMV	4 (2.2)	0	4 (7.7)	0.006
Additional lesions	82 (45.3)	53 (41.1)	29 (55.8)	0.098
Pulmonic valve Z value	− 1.4 (− 2.5, − 0.6)	− 1.3 (− 2.2, − 0.58)	− 1.9 (− 2.9, − 0.9)	0.035
MPA Z value	− 3.1 (− 5.1, − 1.9)	− 3.1 (− 4.8, − 1.8)	− 3.7 (− 6.2, − 2.1)	0.147
RPA Z value	− 0.7 (− 1.5, 0.1)	− 0.6 (− 1.4, 0.1)	− 0.7 (− 1.5, 0)	0.648
LPA Z value	− 0.2 (− 1.2, 0.4)	− 0.1 (− 1.1, 0.6)	− 0.5 (− 1.2, 0.3)	0.352
CPB (min)	139 (110, 165)	130 (107, 160)	149 (130, 187)	0.001
Cross clamp (min)	108 (86, 128)	102 (83, 126)	119 (98, 146)	0.005
Valve preservation	115 (63.5)	92 (71.3)	23 (44.2)	0.001
Residual RVOT max pressure gradient (mmHg)	20 (10, 30)	21 (11, 30)	17 (6, 30)	0.165
Residual VSD	37 (20.4)	25 (19.4)	12 (23.1)	0.684
Residual PR	90 (49.7)	58 (45)	33 (63.5)	0.032
Delayed sternal closure	9 (5)	0	9 (17.3)	< .001
Post-operative ECMO	2 (1)	0	2 (1)	0.025

*SpO*_*2*_ pulse oximetry saturation, *RVOT* right ventricular outflow tract, *max* maximum, *IMV* invasive mechanical ventilation, *MPA* main pulmonary artery, *RPA* right pulmonary artery, *LPA* left pulmonary artery, *CPB* cardiopulmonary bypass, *VSD* ventricular septal defect, *PR* pulmonic regurgitation

^a^For continuous variables, medians (25th percentile, 75th percentile) are presented. For categorical variables, counts (percentages) are presented

^b^Missing data: RVOT max pressure gradient (4), pulmonic valve Z (1), MPA Z (3), RPA Z (1), LPA Z (5)

**Fig. 2:**
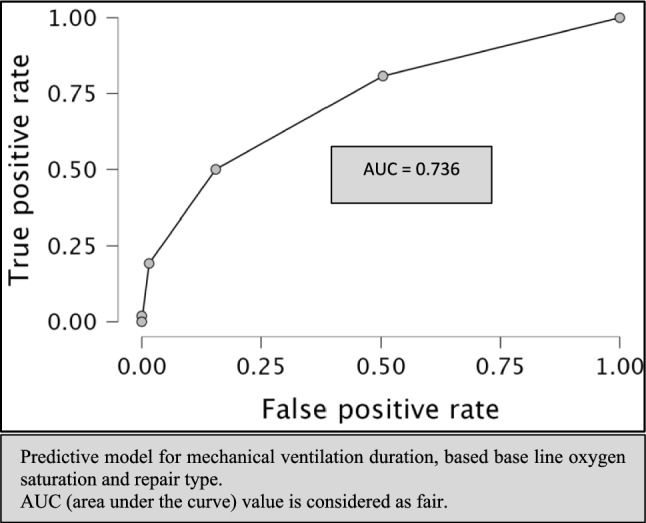
ROC plot

In a multivariate analysis, only baseline SpO_2_ and repair type (valve preservation vs. non-valve-preservation) maintained a statistically significant association with IMV duration, as shown in Table 3. An odds ratio of 0.945 (95% CI 0.919–0.972) for prolonged IMV was associated with baseline saturation (*p* < 0.001). An odds ratio of 0.291 (95% CI 0.143–0.594) was associated with valve-preservation repair (*p* < 0.001). Receiver operating characteristic (ROC) analysis of a prediction model based on baseline saturation and repair type resulted in an area under the curve (AUC) of 0.736, as shown in Fig. 2.

**Table 3:** Multivariate analysis

Variable	OR (95% CI)	*P* value
Pre-operative SpO_2_ (%)	0.945 (0.919–0.972)	< .001
Repair type (valve preservation)	0.291 (0.143–0.594)	< .001

*SpO*_*2*_ pulse oximetry saturation, *OR* odds ratio, *CI* confidence interval

## Discussion

Factors associated with IMV duration post TOF repair were studied in the past and were found to be related to age and size, baseline oxygen saturation, the severity of pulmonary artery stenosis, CPB duration, residual lesions, amount of blood returning to the left atrium on cardiopulmonary bypass and early post-operative ventilator associated pneumonia [5, 7, 24]. Almost all studies directly investigating these factors relate to cohorts of either “standard” timing of repair during infancy or to mixed cohorts of early and late repair after infancy. Isolated forms of TOF were not studied separately. The rationale for our study was to determine whether these risk factors, specifically pre-operative bedside, clinical and echocardiographic markers, apply to our unique TOF population, presenting to medical care and surgical repair at a much older age. We hypothesized that older TOF patients present after a more prolonged exposure of the right ventricle to systemic pressures, causing significant hypertrophy that would incur a more extended period of restrictive physiology, impacting the post-operative period with a longer duration of invasive mechanical ventilation. As no significant previous work had been done to test this hypothesis, we investigated it in our unique population. Therefore, we chose a pragmatic method to correlate easily measured clinical “upstream” factors driving right ventricle hypertrophy to post-operative IMV duration. Our study found that of the variables investigated only baseline oxygen saturation and type of repair were independently associated with IMV duration.

Age and size were equally distributed between the groups, as opposed to previous reports of standard cohorts of repair during infancy, in which younger age and smaller size were associated with prolonged IMV.

Interestingly, while pre-operative SpO_2_ significantly differed between the groups, pre-operative maximum RVOT pressure gradient was neither clinically nor statistically different. The discrepancy might be explained by the fact that pre-operative oxygen saturation more globally reflects the ratio of pulmonary to systemic blood flow (Qp:Qs), in which the degree of RVOT obstruction is only one determinant and is more dependent than perceived, together with the level of the stenosis along the RVOT (sub-valvar, valvar and supra-valvar), the size of the pulmonary annulus and branch pulmonary arteries, systemic vascular resistance, and the effect of a restrictive VSD [25]. This finding is consistent with previous work also showing that higher RVOT pressure gradients were measured in shorter IMV groups, without statistical significance [26].

Four of the patients in the prolonged IMV group were admitted to the PICU and intubated prior to their surgery, secondary to a deep refractory cyanotic spell. One was treated with esmolol infusion prior to surgery, one with phenylephrine, and two with both infusions. The occurrence of spells was the only statistically significant different clinical variable consistent with the reason for intubation. Despite the rarity of refractory hypercyanotic spells, there are previous reports of patients requiring intubation under these circumstances, as well as reports correlating pre-operative intubation with prolonged mechanical ventilation post-operatively [26].

A predictive model based on baseline saturation and repair type (Fig. 2) showed fair accuracy with high specificity (0.938) and low sensitivity (0.308) to prolonged IMV. The practical implication of this knowledge is that late TOF repair patients, with higher baseline saturation and a valve preserving repair would benefit from early extubation with the least exposure to the risks and side effects of extubation failure.

**Fig. 3:**
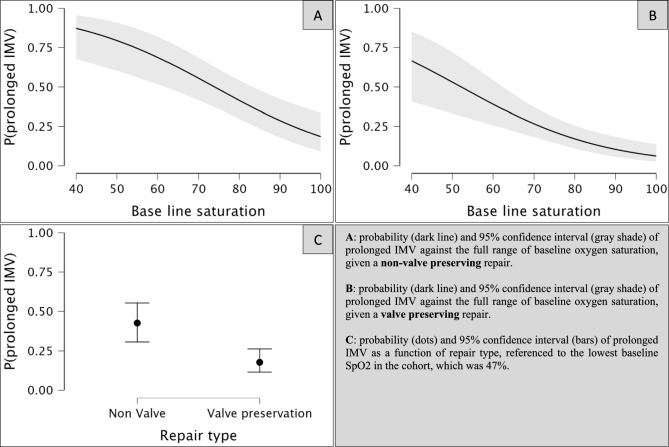
Conditional estimates plots

Figure 3 displays the probability and 95% confidence interval (CI) of prolonged IMV against the full range of baseline oxygen saturation, given a non-valve preserving repair (plot A), a valve preserving repair (plot B), as well as the probability and 95% CI of prolonged IMV as a function of repair type, referenced to the lowest baseline SpO_2_ in the cohort, which was 47% (plot C). Comparing plots A and B shows that a valve preserving repair confers some protection against prolonged IMV given similar baseline saturation values

In an article by Van Ardsell et al. from 2000, the authors describe several changes in the approach to TOF repair in its isolated form, over a 5-year period, based on a cohort of 227 patients, from the timing of primary repair through changes in surgical techniques. In the earlier era described, the median age at repair was above infancy and a bit closer to the age group relevant to our cohort with clinically comparable baseline oxygen saturation, duration of mechanical ventilation, and bypass time. Transannular patch repair was associated with a longer time to extubation in a univariate but not in a multivariate analysis. An extensive comparison to our distinct cohort is limited as surgical techniques have also changed significantly with less utilization of a palliative shunt prior to complete repair, higher efforts to preserve the pulmonary annulus and valve, and the move from trans-ventricular to trans-atrial repair [27].

In a recent large cohort from Indonesia, with a comparable median age to our report, the authors observed that age at repair of less than 50 months, weight of less than 12 kg, duration of CPB, and aortic cross-clamp were associated with prolonged IMV [24]. Our study cohort and its results are unique compared to that and other previous reports. First, by excluding standard repair during infancy, our cohort is highly homogenous and significantly older than previously reported [3–5]. We also excluded the different anatomical variations of TOF, as these present with a different physiology that impacts medical and surgical management. By maintaining the homogeneity of the cohort, our study’s internal and external validity rises. Our study is limited by its design as a retrospective cohort study. As such, it is prone to selection bias and confounding factors that make it unsuited to determine causation despite the found associations. Generalizability is also limited by the fact that this is a single-center cohort. Prolonged duration of IMV is multifactorial. Besides objective physiological considerations, many other factors are important and are related to institutional experience, preferences, and limitations. Reviewing the medical records, we could elicit that the primary reason for delaying extubation in most patients was cardiopulmonary. Further, we believe that the homogeneity of the specific subset of TOF population and the significant statistical results, as well as concordance with previous observations, with respect to baseline SpO_2_ and valve preservation, increase the validity of the results [5, 7].

## Conclusion

Our data show that in distinctly older patients undergoing TOF repair, it is more likely that a longer duration of post-operative IMV will be observed in those with lower baseline SpO_2_ and a non-valve preserving surgery. Other specific clinical markers of the severity of RVOT obstruction were not associated with IMV duration.

## Supplementary Information

Below is the link to the electronic supplementary material.

Supplementary file1 (DOCX 19 kb)

## Data Availability

No datasets were generated or analysed during the current study.

## Abbreviations

TOF: Tetralogy of Fallot
RVOT: Right ventricular outflow tract
VSD: Ventricular septal defect
TAP: Transannular patch
PICU: Pediatric intensive care unit
IMV: Invasive mechanical ventilation
SpO_2_: Pulse oximetry oxygen saturation
ECMO: Extracorporeal membrane oxygenation
MAPCA’s: Major aortopulmonary collaterals
CPB: Cardiopulmonary bypass
