# Supplementary material for: Factors Associated with Prolonged Mechanical Ventilation in Late Repair of Tetralogy of Fallot
Source: Pediatr Cardiol. 2025 Jan 30;47(1):445–52. doi: 10.1007/s00246-025-03786-9 (PMC12827296; doi:10.1007/s00246-025-03786-9)
Supplement: Supplementary file 1 — Supplementary file1 (DOCX 19 kb) [file 246_2025_3786_MOESM1_ESM.docx]

Supplementary Table 1. Additional characteristics

| Variable^a^ | Full cohort  n=181 | Standard IMV  n=129 (71%) | Prolonged IMV  n=52 (29%) | *P* value |
| --- | --- | --- | --- | --- |
| Syndromes | 14 (7.7) | 9 (7) | 5 (9.6) | 0.548 |
| DiGeorge | 2 (1.1) | 1 (0.8) | 1 (2) | 0.263 |
| Trisomy 21 | 4 (2.2) | 4 (3.1) | 0 |  |
| VACTREL | 1 (0.6) | 1 (0.8) | 0 |  |
| Undiagnosed dysmorphism | 7 (3.9) | 3 (2.3) | 4 (7.7) |  |
| Additional pre-operative lesions | 82 (45.3) | 53 (41.1) | 29 (55.8) | 0.098 |
| PFO/ASD | 63 (34.8) | 38 (29.5) | 25 (48.1) | **0.025** |
| Muscular VSD | 4 (2.2) | 3 (2.3) | 1 (1.9) | 0.868 |
| PDA | 8 (4.4) | 5 (3.9) | 3 (5.8) | 0.691 |
| Non-confluent pulmonary arteries | 4 (2.2) | 3 (2.3) | 1 (1.9) | 1 |
| Severe LPA stenosis | 2 (1.1) | 1 (0.8) | 1 (1.9) | 0.493 |
| Coronaries abnormalities | 13 (7.2) | 9 (7) | 4 (7.7) | 1 |
| Interrupted IVC | 1 (0.6) | 1 (0.8) | 0 | 1 |
| Mitral valve prolapse | 1 (0.6) | 1 (0.8) | 0 | 1 |
| Mitral valve cleft | 1 (0.6) | 1 (0.8) | 0 | 1 |
| Right upper pulmonic vein to SVC | 1 (0.6) | 0 | 1 (1.9) | 0.287 |
| Discrete aortopulmonary collaterals | 5 (2.8) | 3 (2.3) | 2 (3.8) | 0.626 |
| Aberrant left subclavian artery | 1 (0.6) | 1 (0.8) | 0 | 1 |
| Left SVC to coronary sinus | 4 (2.2) | 3 (2.3) | 1 (1.9) | 1 |
| Non valve preserving procedures |  |  |  | **0.006** |
| Prosthetic valve | 1 (0.6) | 1 (0.8) | 0 |  |
| TAP | 1 (0.6) | 0 | 1 (1.9) |  |
| TAP + Bicusp | 8 (4.4) | 5 (3.9) | 3 (5.8) |  |
| TAP + Monocusp | 56 (31) | 31 (24) | 25 (48.1) |  |
| ^a^ counts (percentages) are presented.  Abbreviations: VACTREL – syndrome of vertebral anomalies, anal atresia, cardiac anomalies, tracheo-esophageal fistula, esophageal atresia, renal anomalies and limb defects, PFO – patent foramen ovale, ASD – atrial septal defect, VSD – ventricular septal defect, PDA – patent ductus arteriosus, LPA – left pulmonary artery, IVC – inferior vena cava, SVC – superior vena cava. | | | | |
